# Clinical consequences of persistent low level viremia

**DOI:** 10.1186/1742-4690-9-S1-P34

**Published:** 2012-05-25

**Authors:** Toby Dyner, Virginia Cafaro, Valby Chow

**Affiliations:** 1 Hiv Disease at Shared Perspectives On Therapies (Spot), San Francisco, USA

## Introduction

Work done by this group nearly 7 years ago, evaluated a cohort of patients who, for a variety of reasons, elected to remain on a virologically failing ARV regimen. We re-evaluated these patients to determine whether their earlier years living with low level viremia impacted their future treatment options. In the absence of a cure and with the goal of maintaining HIV infection as a chronic, yet manageable disease, it is important to understand the consequences of persistent immune system activation.

## Materials and methods

Two community based HIV practices with nearly 1000 patients evaluated a subset of 25 patients on stable ARV regimens for >24 months who refused treatment change despite the presence of low level viremia. Patients were counseled regarding the need for change. They acknowledged a variety of reasons for their refusal including fear of change and fear of the unknown; a comfort with their current regimen; fear of "burning through options", etc. Charts were reviewed for CD4 counts, HIV bDNA levels, HIV related OIs or malignancies, as well as other co-morbidities.

## Results

As new HIV medications became available, patients agreed to switch regimens. 19 of 25 patients were started on regimens which resulted in virologic suppression below the level of quantification (BLQ). 1 patient died of an MI and 1 died of a neuroendocrine tumor and 4 patients were lost to follow-up. Given the potency of the new regimens that were constructed, those patients who switched were able to suppress without significant difficulties.

## Conclusions

With the new paradigm of test and treat despite CD4 level, we can expect to be treating many more patients for longer periods of time. The issues of adherence and "pill fatigue" are well known and contribute to patients' potential inability to maintain fully suppressive regimens for long and sustained periods of time. Our experience of patients who refused to switch regimens despite low levels of virus and yet, when ready, were able to fully suppress with a regimen change, is encouraging.

